# Real-World Comparison of Human and Software Image Assessment in Acute Ischemic Stroke Patients’ Qualification for Reperfusion Treatment

**DOI:** 10.3390/jcm9113383

**Published:** 2020-10-22

**Authors:** Bartlomiej Lasocha, Roman Pulyk, Pawel Brzegowy, Pawel Latacz, Agnieszka Slowik, Tadeusz J. Popiela

**Affiliations:** 1 University Hospital New Seat (NSSU) Diagnostic Imaging Unit, University Hospital, 30-688 Krakow, Poland; 2 Chair of Neurology, Jagiellonian University Medical College, 31-008 Krakow, Poland; radiologia.cumrik@su.krakow.pl (R.P.); radiologia@su.krakow.pl (P.L.); slowik@cm-uj.krakow.pl (A.S.); 3 Chair of Radiology, Jagiellonian University Medical College, 31-008 Krakow, Poland; neurologia@cm-uj.krakow.pl (P.B.); msjpopie@cyf-kr.edu.pl (T.J.P.)

**Keywords:** stroke, CT perfusion, fibrinolysis, mechanical thrombectomy

## Abstract

Our aim was to compare human and computer accuracy in reading medical images of acute stroke patients. We analyzed data of patients who underwent assessment of Alberta Stroke Program Early CT Score (ASPECTS) and CT Perfusion (CTP) via Rapid Processing of Perfusion and Diffusion (RAPID) software RAPID ASPECTS, and RAPID CTP), compared to radiologist reports and manual measurements. We compared volumes calculated by RAPID CTP software with those selected by scanner-equipped software (GE). For reference, follow-up images were manually assessed in accordance with the Alberta Stroke Program Early CT Score (ASPECTS) territories retrospectively. Although exact ASPECTS score agreement between the automatic and manual methods, and between each method and follow-up, was poor, crossing of the threshold for reperfusion therapy was characterized by an 80% match. CT perfusion analyses yielded only slight agreement (kappa = 0.193) in the qualification of patients for therapy. Either automatic or manual scoring methods of non-contrast images imply similar clinical decisions in real-world circumstances. However, volume measurements performed by fully automatic and manually assisted systems are not comparable. Thresholds devised and validated for computer algorithms are not compatible with measurements performed manually using other software and should not be applied to setups other than those with which they were developed.

## 1. Introduction

Acute ischemic stroke is a leading cause of disability in the western world. Considering the increasing proportion of elderly people in developed countries, we should expect that the associated socioeconomic burden will grow. Given that we have mastered effective reperfusion therapies, it may be that medicine has failed to reverse this trend due strictly to inadequate organization [1]. Early in the advent of fibrinolysis, we learned the limitations of causative treatment strategies, only some of which have been revised in accordance with contemporary techniques of mechanical thrombectomy. Not only was sound evidence limited to large vessel occlusions (LVO) in anterior circulation, but it was also applied six hours from symptom onset only. When cornerstone articles on the widening of time windows were published [2,3], and most guidelines updated [4], a question was raised as to how strictly reperfusion qualification rules should follow those associated with the above-mentioned trials in order to actually replicate their results. A different approach was instituted in the DAWN and DEFUSE 3 studies, the former employing clinical radiological mismatches and the latter comprising purely image-based detection of salvageable tissue, with both using the same particular software. We know that many perfusion-assisted stroke studies were conducted previously, employing numerous software and clinical setups, but the existence of evidence that the amount of viable ischemic brain tissue can be quantified is still arguable [5,6]. This implies another question: what constitutes the difference? Is it the novel—or superior but previously underestimated—algorithm for the assessment of cerebral blood flow parameters, or improvement in the remaining workflow components, such as filtering or segmentation? What is the share of importance in the final results provided by a perfusion examination? Does tissue function expressed by blood circulation give way to post-processing of the data acquired by CT scanners?

There is no doubt that the assistance of machines and software algorithms will progress, eventually replacing human image reading, or at least its most repeatable and time-consuming tasks. However, control of this process requires profound validation and multiple comparisons of results produced by both methods. As opposed to the synthetic studies “in a bubble”—blinded to clinical data, without rush, or even with pre-selected cases—comparing automatic and manual reading [7,8,9], we present a performance summary of heterogeneous radiologist team in a 24/7 service versus fully automatic software solution.

## 2. Experimental Section

A retrospective observational study of acute ischemic stroke patients diagnosed and treated in a tertiary stroke center was performed. The primary outcome was to evaluate how much does automatic and manual interpretation of imaging data differ in produced results, and what circumstances may influence it.

### 2.1. Patient Selection

Each patient with sudden onset neurological deficit, by means of neurological examination diagnosed or suspected of acute ischemic stroke, and who was clinically eligible for reperfusion treatment, was subjected to a multimodal CT scan.

Each patient’s case history was reviewed in order to confirm that the final diagnosis was acute ischemic stroke, rather than a stroke mimic. We excluded patients whose inability to cooperate resulted in movement artifacts rendering all or parts of the imaging study unacceptable. Furthermore, we excluded cases in which regions of interest (ROIs) for arterial input or venous outflow curves of the perfusion scan could not be found or were unsatisfactory, as well as cases in which the Rapid Processing of Perfusion and Diffusion (RAPID) system failed to process data. We required completeness of imaging. Accordingly, cases with massive hemorrhagic transformation, or those lost to follow-up due to having been transferred to other hospitals (thus, preventing follow-up assessment) were rejected. Since the scores we use and trials we refer to focus on anterior circulation large vessel occlusions, the vertebrobasilar system, distal, and lacunar ischemic strokes were not included.

The first hundred patients subjected to a stroke imaging protocol that included automatic RAPID assessment, and who met all the requirements, were analyzed.

### 2.2. Image Acquisition and Analysis

All of the imaging was performed on a 64-slice scanner (GE Optima 660) located in the emergency department. Imaging workup comprised a non-contrast head examination (non-contrast CT, or NCCT), multiphase CT angiography (mCTA) of the head and neck, and perfusion CT (pCT) of the brain. If intravenous treatment was instituted, the bolus was given between the NCCT and mCTA procedures, and infusion was initiated so as to be completed during following procedures. The details of imaging protocol are presented in Appendix A.

Upon acquisition, the images were transferred to workstations, and the Picture Archiving and Communication System (PACS). On-duty radiologists assessed NCCT for the presence of bleeding or any pathology other than ischemia that might present as a stroke. When both of these possibilities had been excluded, the images were scored using the Alberta Stroke Program Early CT Score (ASPECTS) scale and the results included in the report. The mCTA results were assessed for the presence of large vessel occlusion (LVO), hemodynamically significant stenosis, and, additionally, for collateral scoring; however, the last-mentioned item was not included in the current analysis.

Perfusion studies were processed using workstations supplied by the scanner’s manufacturers (GE Advantage 4.6, software version 11.3 ext2). Parametric maps of cerebral blood volume (CBV), cerebral blood flow (CBF), mean transit time (MTT), and time to maximum (TMAX) were calculated. Using this software, the user is able to set a range or threshold for any generated map and calculate the respective volume; the threshold, however, must be absolute. It is possible to define a region of interest (ROI) on any particular map, transfer it to other maps, and mirror it to the opposite side in order to calculate measurements in relation to “healthy” tissue. However, the software does not enable the user to define a segment according to its relationship with values of the other side, as, for example, below 30% [10] of the contralateral hemisphere. It is not possible to calculate the volume of tissue fulfilling the criteria as thus defined. The Institution employed thresholds of CBV below 1 mL/100 g [11] and MTT values above 10 s [12] for definitions of infarct core and penumbra respectively. A core volume exceeding one-third of the penumbra was considered a contraindication for reperfusion therapy [3], however patients fulfilling criteria of either DEFUSE 3 or DAWN were treated accordingly to these protocols. The generated maps, with a table of measurements and examples of defined ROIs, were also appended to each patient’s baseline exam and stored in the PACS.

The non-contrast and perfusion scans were additionally transferred to the RAPID system providing automatic ASPECTS scoring and analysis of perfusion source images with respect to DEFUSE 3 criteria. The results were sent back to the institutional PACS as additional images appended to the exam in question.

In controversial cases, the decision regarding reperfusion treatment was made by the coordinating neurologist and interventional neuroradiologist. These were mainly rapidly progressing ischemia with extensive core within time window of six hours, discrepancies between ASPECTS and perfusion studies, or between the assessment of the same parameter by radiologist and RAPID system. Less frequently, a tailored treatment was caused by tandem or bilateral lesions, complex anatomy, and comorbidities.

Since the software supplied by GE (the scanner vendor) did not enable users to calculate maps of a parameter in relation to the other side, TMAX volume was the only measurement suitable for direct “man-to machine” comparison. We also performed comparison of volumes for penumbra as defined by RAPID and by our local routine.

Each of the manually generated maps was assessed using the ASPECTS approach, and each lesion exceeding either our institutional thresholds or those used in DEFUSE 3 was recorded as perfusion ASPECTS (pASPECTS). Only in the case of the CBF map did we inspect the images and score them when a lesion was apparent, since we were unable to apply a numerical criterion in relation to the other side.

For the purposes of the study, follow-up CT was reviewed retrospectively in order to apply ASPECTS scoring and define the territories involved.

### 2.3. Statistical Analysis

The Shapiro–Wilk test was used to asses a normal distribution of numeric features. For continuous variables of normal distribution, means and standard deviations are presented; for ordinal and non-normal distributed continuous variables, medians and interquartile ranges are given. Relations between variables were estimated using Pearson’s r for continuous variables and Spearman’s rho for ordinal variables. Consistency between methods was measured by percentage agreement and Cohen’s kappa in categorical and ordinal scores, and by interclass coefficient [13] in continuous variables. Bland–Altman plots were sketched to visualize agreement ranges and patterns [14]. Differences between groups were assessed by means of the Wilcoxon test. A *p*-value below 0.05 was adopted as a statistical significance threshold.

We performed the tests, calculated the measures and indices, and plotted the diagrams using R Software version 3.6.2 [15].

## 3. Results

During the period 1 January–1 July, 2019, 283 patients suspected of having experienced acute ischemic stroke underwent imaging. Of the results, 49 imaging datasets were incomplete or invalid for the purposes of the study; in 62 patients, acute ischemic stroke was ruled out following tests or clinical histories. In 72 patients, the pathology turned out to be either non-large vessel occlusion (non-LVO) or posterior circulation ischemia. The demographics of the enrolled patients is presented in Table 1.

### 3.1. Non-Contrast CT

#### 3.1.1. Method and Score Related Variability

The global follow-up ASPECTS result was lower than the baseline (see Supplementary Materials Figures S1 and S2). We proved the statistical significance of this shift in both methods of baseline assessment (*p*-value = 0.016 for manual and 0.032 for automatic assessment respectively).

This tendency was absent in two subgroups: patients treated intravenously when their ASPECTS scores were analyzed automatically, and patients who underwent combined treatment when assessed manually (see Tables S1 and S2 in the Supplementary Materials).

The numerical agreement of the baseline and follow-up ASPECTS scoring exhibited a fair degree of result matching when minimal tolerance margins were applied and when analyzed field-by-field. Agreement was slightly better for manual methods, but RAPID was shown to be superior when agreement corrected for chance was analyzed. Both methods exhibited a significant reduction in degree of matching for exact results.

When the question was narrowed down to, “ASPECTS less than 6, versus 6 and above,” agreement was almost identical for the radiologist on duty and software algorithms. Finally, a closer correlation with follow-up imaging was observed for manual assessment than for RAPID ASPECTS [16] (Table 2).

In-depth analysis of particular ASPECTS scale territories showed the closest agreement with follow-up CT in lentiform nuclei. Both methods achieved the highest kappa coefficients, which, in the case of manual scoring, were as high as 0.86.

On the other hand, this analysis revealed the weaknesses of the ASPECTS approach in posterior areas of the cortex supplied by the middle cerebral artery and at the supra-ganglionic level. The most prominent examples were the M3 and M5 territories, where RAPID achieved its lowest (0.2) and manual assessment its second-lowest (0.38) level of agreement.

The worst mismatch between methods was observed in the case of the caudate nucleus, where the accuracy of automatic detection was substantially lower, whereas the overall lesion count in this region was in line with both manual and follow-up (Table 3 and Table 4).

#### 3.1.2. Patient and Rater Related Variability

Analysis of the influence of patient clinical features on ASPECTS scores measured both automatically and manually revealed a decrement in general scores along with increases in the National Institutes of Health Stroke Scale (NIHSS), irrespective of the method used. However, only manual assessment was bound to the time between symptom onset and CT. A negative correlation was also found between NIHSS values and the discrepancy between manual baseline assessment and follow-up (*p* = 0.004, see Tables S3 and S4 in the Supplementary Materials).

Differences were noted in the performance of raters; however, neither number of years of experience (*p*-value = 0.266) nor workload (*p*-value = 0.515) influenced the accuracy of assessment. When the score results were compared to the threshold of 6, the calculated agreement was fair to perfect (55–100%); when each field of the ASPECTS grading system was compared separately, agreement was good to perfect (67–90%) [17]. Again, a strikingly low level of agreement was observed for exact global ASPECTS scores (0–33%) (Table 5); however, for the raters yielding the lowest results, an additional comparison was made with automatic data analysis of the very same cases, showing more or less the same degree of accuracy (Supplementary Materials Table S5).

### 3.2. CT Perfusion

#### 3.2.1. Measurements of Lesion Volumes

Proceeding to perfusion studies, a discrepancy is visible at first glance in volumes measured by automated vs. manual methods (Table 6). In each patient, they are incommensurable. However, for the study population as a whole, when using follow-up ASPECTS as a reference, affected tissue quantities proved to be correlated with final necrosis area. The closest connection of this type was observed for automatic detection of decreased CBF, followed by elongated TMAX and manually assessed CBV. The weakest correlation (below the level of statistical significance) was observed for manual assessment of TMAX and MTT lesions.

While infarct estimate volumes in our study were far from equal (interclass coefficient (ICC) = 0.17, *p*-value = 0.102), they were closely correlated with each other (details in Supplementary Materials Table S6). Nevertheless, reperfusion qualification based on RAPID results following DEFUSE 3 criteria overlapped with our institutional routine only in 63 percent, and following DAWN in 55 (see Supplementary Table S7).

Hypoperfusion determinants, i.e., RAPID TMAX and manual MTT volumes, were the most distantly correlated measurements in our study, with correlations of only 0.292 (*p*-value = 0.004), and no agreement between results (ICC = 0.054, *p*-value = 0.199).

Manual vs. RAPID TMAX > 6 s volume measurement was, on average, more than twofold higher in manual assessment; proof of agreement was lacking.

The last parameter, mismatch ratio, also failed to exhibit any consistency between automatic and manual methods. Moreover, even the correlation between them was doubtful (*p*-value = 0.251).

#### 3.2.2. Perfusion ASPECTS

Applying the ASPECTS approach to perfusion studies, when compared to non-contrast CT, pASPECTS accuracy was inferior. Through field-by-field analysis, we learned that CBF and CBV ASPECTS performed similarly to plain CT studies, but in the cases of MTT and TMAX, the results were significantly worse. Agreement with baseline imaging results was no better. It is worth mentioning, however, that scores derived from maps of CBF reflected final tissue injury more accurately than the others (kappa = 0.321, absolute agreement 30%) (Table 7).

Additionally, we found substantial differences in agreement between baseline pASPECTS and follow-up ASPECT, depending on treatment strategy. In patients with no causative treatment, TMAX ASPECTS agreed most closely with the final result, whereas in IV-thrombolysis-only patients CBF ASPECTS produced better results. In all patients undergoing mechanical thrombectomy, CBV ASPECTS performance was unsurpassed, while MTT maps and its respective ASPECTS scores proved to be least useful. Again, in most cases, the follow-up imaging revealed even more extensive ischemic areas than those expected via penumbra (Figures S3–S5 and Tables S8–S12 in Supplementary Materials).

## 4. Discussion

Neither manual nor automatic assessment proved to be superior in ASPECTS scoring. Moreover, in clinical practice, they imply even more convergent decisions. Perfusion analysis however, exhibits issues of each method, with manual approach coming off a bit short. The most striking, however, is the fact that patients get worse despite therapy. Even in cases in which causative treatment was instituted, comparisons between baseline and follow-up exhibit progression.

This is not a new finding [18], although it has not yet been directly addressed. Recently, very convincing studies have been published claiming that ASPECTS overestimates tissue injury and that early CT lesions, if promptly treated, may resolve [19,20]. Assuming these lesions are associated with ionic edema [21], this can be only partially true; our study provides data concerning this question. Some of these phenomena may be attributed to selection bias. Firstly, the subgroup of patients disqualified from reperfusion exhibited the closest match between follow-up and TMAX pASPECTS, which is a penumbra marker. This is explained by the evolution of penumbra to infarction. We confirmed that reperfusion may reduce this shift to the level of statistical randomness. Secondly, patients qualified for thrombolysis or thrombectomy were assumed to have been imaged earlier in the course of their disease; therefore, the lesions, although inevitable, were not yet visible. Whereas, for core markers, underestimation can be explained by the time elapsed from imaging to recanalization, we are uncertain why the same shift occurs between penumbra maps and follow-up. Similar phenomena have been explained to date by edema and partial or delayed recanalization [22] alone; this may by supported by our observation, since the progression occurs in all subgroups, except “no reperfusion.” One explanation may involve reperfusion injury itself; another may involve the interim character of perfusion studies in general, although this is not a low-sensitivity or -specificity issue.

The low level of agreement in exact scores can easily be explained by the intrinsic nature of the ASPECTS score and agreement test; one in ten field results disagree, but tolerance-adjusted results prove a more generalized convergence.

It would be desirable for particular maps to enable us to identify lesions that are unequivocal on plain CT, thus, overcoming the anatomic and physiologic limitations of NCCT ASPECTS. Our results support the finding that perfusion ASPECTS scores are related to baseline, follow-up, and to each other. We did not expect to find that perfusion ASPECTS had no advantage at all over NCCT ASPECTS in predicting the extent of final necrosis. Previously reported results, although promising, were not decisive [6], and were correlated more closely with clinical outcome, something that cannot be said for CT ASPECTS [11,23].

The discrepancy in exact results for perfusion volumes was not surprising, as this has already been reported [24,25]; however, we did not expect to find disagreement on such a scale. Due to the distribution abnormalities of volumetric parameters, development of regression models and alternative (model-derived) qualification thresholds would not produce reasonable results.

First of all, a difference in deconvolution algorithms exists between the two software packages: GE uses Singular Value Decomposition plus (SVD+), whereas RAPID uses block-circulant deconvolution (bSVD) [26]. It had been found previously [27], which the two algorithms produce divergent results in the manner we experienced; however, this was expressed in the perfusion values measured, not in the extent of tissue damage. This observation may also be explained by differences in filtering, motion correction, and segmentation, which were presumably more advanced in RAPID software, as the images upon visual inspection were smoother and less noisy, but characterized by greater contrast (Figure S6 in Supplementary Materials), and the marked areas avoided fluid spaces and blood vessels.

Another interesting observation to comment on is that, in a group of thrombolysed patients, RAPID ASPECTS and CBF—a RAPID CTP-assessed parameter [28]—pASPECTS performed best in predicting the ultimate area of brain damage. The software was validated in a thrombectomy trial (DEFUSE 2), however, datasets used to develop RAPID software were acquired in an intravenous (i.v.) fibrinolysis (DEFUSE 1) study [2,29], what might result in fine-tuning algorithms to this specific setup. Finally, it must be stressed that the map produced by the RAPID system was proven superior in predicting the area of final tissue damage.

Our study is characterized by certain drawbacks. It involves a single center and is retrospective. The sample size is not impressive; nevertheless, it is characterized by sufficient statistical power for most of our analyses. Moreover, accepting follow-up ASPECTS as a reference imposes several limitations. Firstly, a global ASPECTS score is only roughly an ordinal biomarker, since neither the eloquence nor the volume of the fields are equally distributed. Secondly, it is image-based and may not reflect the patient’s clinical condition. Thirdly, neither the side nor the function of affected territories produces changes in the score. Fourthly, ASPECTS is more of a predictive than a prognostic score [30], and its features, when applied to follow-up examination, have not been studied heretofore. Fifthly, follow-up ASPECTS was assessed manually, and was prone to the same vulnerabilities as manual baseline assessment. On the other hand, using an imaging biomarker has the great advantage of reproducibility, enabling not only comparisons but also scaling of the method to an unlimited number of cases. The score itself has been proved reliable, and is widely known. It is not as volatile as clinical features, which may not be available for assessment at a later time, and thus enables numerous time-independent assessments. Moreover, whereas acute stroke assessment may result in some hesitation, at follow-up the demarcation of infarcted tissue renders the scoring process almost mechanical.

## 5. Conclusions

There are no convincing reasons to reject ASPECTS scoring. Neither a low level of experience nor a heavy workload impaired the quality of assessment compared to follow-up studies or automatic methods. Perfusion ASPECTS does not improve qualification results when compared to plain CT. Measurement discordances between software vendors prevent the universal adoption of perfusion CT thresholds, although multiple clinical trials have made use of it. ASPECTS scores in the majority of cases drop over time even if reperfusion therapy is immediate. Imaging deterioration cannot be predicted by perfusion ASPECTS, but this may differ between specific diagnostic and therapeutic setups.

## Figures and Tables

**Table 1:** Study population.

Demographic Characteristic	Study Group *n* = 100
Female	59 (59%)
Age	73 (66–83)
Hypertension	82 (82%)
Atrial fibrillation	34 (34%)
Diabetes	28 (28%)
NIHSS *	17 (9–20)
Time to CT	233 (142–291)
Thrombolysis	53 (53%)
Thrombectomy	49 (49%)

* NIHSS—National Institutes of Health Stroke Scale.

**Table 2:** Comparison of agreement between Alberta Stroke Program Early CT Score (ASPECTS) scoring methods.

NCCT ASPECTS		Manual	RAPID	*p*-Value *
Total score average (standard deviation)		6.84 (2.76)	6.7 (3.01)	n/a
Single territory agreement	Percentage	79%	76%	0.16
	kappa	0.55	0.5	0.16
Score absolute agreement	Percentage (tolerance = 0)	16%	26%	0.99
	Percentage (tolerance = 2)	79%	71%	0.99
	kappa	0.44	0.47	0.99
Correlation	rho	0.71	0.66	n/a
Score relative (<6) agreement	Percentage	80%	78%	0.73
	kappa	0.47	0.45	0.73

* Refers to Cohen’s kappa.

**Table 3:** Performance (in relation to follow-up) of single-field manual ASPECTS assessment.

Territory	Agreement		Lesion Frequency (Baseline)	Lesion Frequency (Follow-Up)
	Percentage	kappa		
CN	89%	0.76	36%	37%
LN	84%	0.86	51%	49%
IC	74%	0.46	35%	43%
IR	77%	0.5	65%	66%
M1	85%	0.59	20%	27%
M2	78%	0.56	38%	50%
M3	74%	0.31	13%	33%
M4	83%	0.51	15%	28%
M5	69%	0.38	27%	50%
M6	77%	0.46	16%	39%

CN = caudate nucleus, LN = lenticular nucleus, IC = internal capsule, IR = insular ribbon, M1–M6 = respective areas of middle cerebral artery supply.

**Table 4:** Performance (in relation to follow-up) of single-field Rapid Processing of Perfusion and Diffusion (RAPID) ASPECTS assessment.

Territory	Agreement		Lesion Frequency (Baseline)	Lesion Frequency (Follow-Up)
	Percentage	kappa		
CN	76%	0.49	37%	37%
LN	84%	0.68	49%	49%
IC	79%	0.56	34%	43%
IR	76%	0.51	56%	66%
M1	80%	0.49	27%	27%
M2	79%	0.58	37%	50%
M3	74%	0.38	25%	33%
M4	85%	0.6	21%	28%
M5	60.0%	0.2	18%	50%
M6	71.0%	0.35	26%	39%

CN = caudate nucleus, LN = lenticular nucleus, IC = internal capsule, IR = insular ribbon, M1–M6 = respective areas of middle cerebral artery supply.

**Table 5:** Agreement on ASPECTS assessment by individual rater, sorted by number of examinations included.

Rater Number	Years of Experience	Number of Patients	Single-Field			Score				Relative to 6		
			Percentage Agreement	kappa	*p*	Percentage Agreement		kappa	*p*	Percentage Agreement	kappa	*p*
						T = 0	T = 2					
1	17	1	90%	0.74	0.16	0%	100%	0	n/a	100%	n/a	n/a
2	10	5	82%	0.63	<0.001	20%	80%	0.090	0.500	100%	1	0.025
3	18	6	83%	0.45	<0.001	33%	83%	0.226	0.100	100%	n/a	n/a
4	16	7	81%	0.51	<0.001	14%	86%	0.106	0.087	100%	n/a	n/a
5	28	7	83%	0.65	<0.001	14%	100%	0.087	0.290	71%	0.36	0.212
6	14	8	80%	0.56	<0.001	0%	88%	−0.160	0.686	100%	1	0.005
7	6	9	81%	0.61	<0.001	11%	89%	0.137	0.126	100%	1	0.003
8	8	11	77%	0.52	<0.001	27%	64%	0.154	0.129	55%	0	n/a
9	23	12	67%	0.33	<0.001	17%	58%	0.055	0.578	67%	0.31	0.276
10	10	16	81%	0.62	<0.001	19%	75%	0.100	0.205	75%	0.39	0.051
11	11	18	79%	0.54	<0.001	11%	83%	−0.011	0.900	72%	0.151	0.457
Junior (≤10 years of experience)		41	80%	0.59	<0.001	20%	76%	0.112	0.022	73%	0.47	<0.001
Senior (>10 years of experience)		59	78%	0.52	<0.001	14%	81%	0.027	0.55	73%	0.379	0.004

**Table 6:** Volumes of perfusion lesions measured via automatic and manual methods.

Map/Method	Median (IQR)	Min–Max	Respective Map ASPECTS		Follow-Up ASPECTS	
			Correlation: rho	Correlation: *p*-Value	Correlation: rho	Correlation: *p*-Value
CBF/RAPID	9 (0–40)	0–169	**−0.47**	**<0.001**	**−0.686**	**<0.001**
TMAX/RAPID	125 (65.5–179)	0–582	**−0.535**	**<0.001**	**−0.486**	**<0.001**
TMAX/Manual	331 (254–385)	125–503	**−0.363**	**<0.001**	−0.114	0.262
MTT/Manual	356 (288–415)	176–542	**−0.244**	**0.015**	−0.060	0.556
CBV/Manual	95 (62–152)	18–333	**−0.285**	**0.004**	**−0.236**	**0.019**

IQR—Inter Quartile Range, CBF—Cerebral Blood Flow, TMAX—Time to Maximum, MTT—Mean Transit Time, CBV—Cerebral Blood Volume, RAPID—Rapid Processing of Perfusion and Diffusion, a software suite for automatic image analysis in acute stroke setting. Bold values indicate *p*-values below 0.005.

**Table 7:** Global perfusion ASPECTS score and relation to non-contrast imaging.

Map	Follow-Up ASPECTS				Baseline RAPID ASPECTS				Baseline Manual ASPECTS			
	Agreement		kappa	*p*-Value	Agreement		kappa	*p*-Value	Agreement		kappa	*p*-Value
	T = 0	T = 2			T = 0	T = 2			T = 0	T = 2		
CBV	22%	73%	0.278	<0.001	31%	76%	0.334	<0.001	39%	85%	0.525	<0.001
CBF	30%	72%	0.321	<0.001	19%	56%	0.174	0.014	19%	59%	0.155	0.006
MTT	18%	57%	0.198	<0.001	14%	41%	0.102	0.078	18%	42%	0.099	0.063
TMAX	22%	64%	0.294	<0.001	20%	49%	0.172	0.004	10%	46%	0.065	0.249

## Supplementary Materials

The following are available online at https://www.mdpi.com/2077-0383/9/11/3383/s1, Figure S1: Bland-Altman plot of manual versus follow-up ASPECTS, Figure S2: Bland-Altman plot of automatic vs. follow-up ASPECTS, Figure S3: Bland-Altman plot of manual CBV < 1 mL/100 g versus RAPID rCBF < 30% volumes, Figure S4: Bland-Altman plot of manual TMAX > 6 s versus RAPID TMAX > 6 s volumes, Figure S5: Bland-Altman plot of manual MTT > 10 s versus RAPID TMAX > 6 s volumes, Figure S6: Different software produces different maps: RAPID CBF map (left); GE perfusion 4 D CBF map (right) Table S1: Reperfusion therapy impact on manual vs. follow-up ASPECTS, Table S2: Reperfusion therapy impact on automatic versus follow-up ASPECTS, Table S3: Correlation between ASPECTS and NIHSS, Table S4: Correlation between ASPECTS and time from symptom onset to CT, Table S5: Agreement of global RAPID ASPECTS scores relative to 6 for raters with percentages of agreement equal or less than 75%, Table S6: Agreement and correlation between perfusion volumes acquired automatically and manually, Table S7: Qualification results comparison chart, Table S8: Reperfusion therapy impact on CTP concordance with follow-up, Table S9: Reperfusion therapy impact on CBF ASPECTS versus follow-up ASPECTS, Table S10: Reperfusion therapy impact on CBV ASPECTS versus follow-up ASPECTS, Table S11: Reperfusion therapy impact on MTT ASPECTS versus follow-up ASPECTS, Table S12: Reperfusion therapy impact on TMAX ASPECTS versus follow-up ASPECTS.

## Appendix A

For NCCT, an axial algorithm was used, utilizing collimation slices 20 mm thick, with a rotation time of 2 s. The X-ray tube potential was set to 120 kV; the anode current was 215 mA. A 25-cm circular field of view (FOV) was reconstructed from the 32 cm data acquisition area, with slice thicknesses of 2.5 and 1.25 mm.

CT angiography employed the helical mode, with total collimation of 40 mm, table feed of 39.375 mm, and rotation time of 0.5 s. The X-ray tube potential was 120 kV. Anode current was set to 320 mA. A 27.4-cm circular FOV was reconstructed from the 32-cm data acquisition area, with a slice thickness of 0.625 mm. The scan was triggered by a ROI placed on the aortic arch; 80 mL of iodine contrast agent was injected into the antecubital vein, followed by 40 mL of saline. The study consisted of four successive phases, the first covering the head and neck, the remainder—the head only. Temporal resolution of the study was 8 s.

CT perfusion was performed using the axial mode, with total collimation of 40 mm and rotation time of 0.8 s. The X-ray tube potential was 80 kV; the anode current was set to 125 mA. A 22 cm circular FOV was reconstructed from the 32 cm data acquisition area, producing eight slices of 5 mm each. Forty-five ml of contrast agent was infused into the antecubital vein, followed by 24 mL of saline flush. The scan was conducted for 30 s, producing 37 time points. To cover a sufficient area of the brain, two consecutive acquisitions were performed, one above the other, 5 mm apart, separated by 2 min to allow for clearance of the contrast media.

A follow-up examination was performed 24 h after reperfusion therapy or baseline imaging in cases where neither rTPA nor thrombectomy was instituted. The scan parameters were very similar to initial acquisition: axial mode, collimation 20 mm, rotation time 2 s. The X-ray tube potential was set at 120 kV; the anode current was 138 mA. A 25-cm circular FOV was reconstructed from the 32 cm data acquisition area, with slice thicknesses of 2.5 and 1.25 mm.

## References

[1] Papanagiotoi P., Ntaios G. (2018). Endovascular thrombectomy in acute ischemic stroke. Circ. Cardiovasc. Interv..

[2] Jovin T.G., Saver J.L., Ribo M., Pereira V., Furlan A., Bonafe A., Baxter B., Gupta R., Lopes D., Jansen O. (2017). Diffusion-weighted imaging or computerized tomography perfusion assessment with clinical mismatch in the triage of wake up and late presenting strokes undergoing neurointervention with Trevo (DAWN) trial methods. Int. J. Stroke.

[3] Albers G.W., Langsberg M.G., Kemp S., Tsai J.P., Lavori P., Christensen S., Mlynash M., Kim S., Hamilton S., Yeatts S.D. (2017). A Multicenter randomized controlled trial of endovascular therapy following imaging evaluation for ischemic stroke (DEFUSE 3). Int. J. Stroke.

[4] Powers W.J., Rabinstein A.A., Ackerson T., Adeoye O.M., Bambakidis N.C., Becker K., Biller J., Brown M., Demaerschalk B.M., Hoh B. (2019). Guidelines for the early management of patients with acute ischemic stroke: 2019 update to the 2018 guidelines for the early management of acute ischemic stroke: A guideline for healthcare professionals from the American Heart Association/American Stroke Association. Stroke.

[5] Borst J., Berkhemer O.A., Ross Y.B., Van Bavel E., van Zwam W.H., van Oostenbrugge R.J., van Walderveen M.A., Lingsma H.F., van der Lugt A., Dippel D.W. (2015). Value of computed tomographic perfusion-based patient selection for intra-arterial acute ischemic stroke treatment. Stroke.

[6] Campbell B.C., Yasssi N., Ma H., Sharma G., Salinas S., Churilov L., Meretoja A., Parsons M.W., Desmond P.M., Lansberg M.G. (2015). Imaging selection in ischemic stroke: Feasibility of automated CT-perfusion analysis. Int. J. Stroke.

[7] Albers G.W., Wald M.J., Mlynash M., Endres J., Bammer R., Straka M., Maier A., Hinson H.E., Sheth K.N., Taylor Kimberly W. (2019). Automated calculation of Alberta Stroke Program Early CT Score: Validation in patients with large hemispheric infarct. Stroke.

[8] Herweh C., Ringleb P.A., Rauch G., Gerry S., Behrens L., Möhlenbruch M., Gottorf R., Richter D., Schieber S., Nagel S. (2016). Performance of e-ASPECTS software in comparison to that of stroke physician on assessing CT scans of acute ischemic stroke patients. Int. J. Stroke.

[9] Nagel S., Sinha D., Day D., Reith W., Chapot R., Papanagiotou P., Warburton E.A., Guyler P., Tysoe S., Fassbender K. (2017). e-ASPECTS software is non inferior to neuroradiologists in applying the ASPECT score to computed tomography scans of acute ischemic stroke patients. Int. J. Stroke.

[10] d’Estere C.D., Boesen M.E., Hwan S., Pordeli P., Najm M., Minhas P., Davari P., Fainardi E., Rubiera M., Khaw A.V. (2015). Time-dependent computed tomographic perfusion thresholds for patients with acute ischemic stroke. Stroke.

[11] Padroni M., Bernardon A., Tamborino C., Roversi G., Borrelli M., Saletti A., De Vito A., Azzini C., Borgatti L., Marcello O. (2019). Cerebral blood volume ASPECTS is the best predictor of clinical outcome in acute ischemic stroke: A retrospective, combined semi-quantitative and quantitative assessment. PLoS ONE.

[12] Vilela P., Rowley H.A. (2017). Brain ischemia: CT and MRI techniques on acute ischemic stroke. Eur. J. Radiol..

[13] Watson P.F., Petrie A. (2010). Method agreement analysis: A review of correct methodology. Theriogenology.

[14] Bland M.J., Altman D.G. (1999). Measuring agreement in method comparison studies. Stat. Methods Med. Res..

[15] R Core Team (2019). R: A Language and Environment for Statistical Computing.

[16] Rapid ASPECTS. https://www.rapidai.com/rapid-aspects.

[17] Morgan C.J., Aban I. (2016). Methods for evaluating the agreement between diagnostic tests. J. Nucl. Cardiol..

[18] Vanicek J., Cimflova P., Bulik M., Jarkovsky J., Prelecova V., Szeder V., Volny O. (2019). Single-centre experience with patients selection for mechanical thrombectomy based on automated computed tomography perfusion analysis—A comparison with computed tomography ct perfusion thrombectomy trials. J. Stroke Cerebrovasc. Dis..

[19] Kaesmacher J., Chalouolos-Iakovidis P., Panos L., Mordasini P., Michel P., Hajdu S.D., Ribo M., Requena M., Maegerlein C., Friedrich B. (2019). Mechanical thrombectomy in ischemic stroke patients with alberta stroke program early computerd tomography score 0–5. Stroke.

[20] Kleine J.F., Kaesmacher M., Wiestler B., Kaesmacher J. (2017). Tissue-selective salvage of the white matter by successful endovascular stroke therapy. Stroke.

[21] Von Kummer R., Dzialowski I. (2017). Imaging of cerebral ischemic edema and neuronal death. Neuroradiology.

[22] Man F., Patrie J.T., Xin W., Zhu G., Hou Q., Michel P., Eskandari A., Jovin T., Xian J., Wang Z. (2015). Delay-sensitive and delay-insensitive deconvolution perfusion-CT: Similar ischemic core and penumbra volumes if appropriate threshold selected for each. Neuroradiology.

[23] Demeestere J., Scheldmann L., Cornelissen S.A., Heye S., Wouters A., Dupont P., Christensen S., Mlynash M., Albers G.W., Lansberg M. (2018). ASPECTS versus CT perfusion to predict functional outcome after successful reperfusion in acute ischemic stroke. Stroke.

[24] Xiong Y., Huang C.C., Fisher M. (2019). Comparison of automated CT perfusion softwares in evaluation of acute ischemic stroke. J. Stroke Cerebrovasc. Dis..

[25] Austein F., Riedel C., Kerby T., Meyne J., Binder A., Lindner T., Huhndorf M., Wodarg F., Jansen O. (2016). Comparison of perfusion CT software to predict the final infarct volume after thrombectomy. Stroke.

[26] Straka M., Albers G.W., Bammer R. (2010). Real-time diffusion-perfusion mismatch analysis in acute stroke. J. Magn. Reson. Imaging.

[27] Kudo K., Sasaki M., Yamada K., Momoshima S., Utsunomiya H., Shirato H., Ogasawara K. (2010). Differences in CT perfusion maps generated by different commercial software: Quantitative analysis by using identical source data of acute stroke patients. Radiology.

[28] Rapid CTP. https://www.rapidai.com/rapid-ctp.

[29] Albers G., Thijs V.N., Wechsler L., Kemp S., Schlaug G., Skalabrin E., Bammer R., Kakuda W., Lansberg M.G., Shuaib A. (2006). Magnetic resonance imaging profiles predict clinical response to early reperfusion: The diffusion and perfusion imaging evaluationfor understanding stroke evolution (DEFUSE) study. Ann. Neurol..

[30] Ntaios G., Papavasileiou V., Michel P., Tatlisumak T., Strbian D. (2015). Predicting functional outcome and symptomatic intracranial hemorrhage in patients with acute ischemic strokea glimpse into the crystal ball?. Stroke.

