# Supplementary material for: Real-World Comparison of Human and Software Image Assessment in Acute Ischemic Stroke Patients’ Qualification for Reperfusion Treatment
Source: J Clin Med. 2020 Oct 22;9(11):3383. doi: 10.3390/jcm9113383 (PMC7690255; doi:10.3390/jcm9113383)
Supplement: Supplementary file 1 [file jcm-09-03383-s001.zip › supplementary materials 3/Table S1.docx]

**Table S1.** Reperfusion therapy impact on manual vs follow-up ASPECTS

| Manual ASPECTS assessment | | | | |
| --- | --- | --- | --- | --- |
| Reperfusion | Agreement | | kappa | U-test  p-value |
|  | t = 0 | t = 2 |  |  |
| No reperfusion | 9% | 83% | 0.067 | < .001 |
| Thrombectomy | 12% | 76% | 0.205 | .002 |
| Fibrinolysis | 21% | 81% | 0.369 | .009 |
| Fibrinolysis and thrombectomy | 12% | 80% | 0.157 | .104 |
| Thrombectomy without fibrinolysis | 13% | 71% | 0.299 | .006 |
| Fibrinolysis without thrombectomy | 29% | 82% | 0.411 | .001 |
| Overall | 16% | 79% | 0.291 | < .001 |

In all cases of statistically significant differences, a negative shift was detected: follow-up ASPECTS scores were lower than baseline.
