# Supplementary material for: Real-World Comparison of Human and Software Image Assessment in Acute Ischemic Stroke Patients’ Qualification for Reperfusion Treatment
Source: J Clin Med. 2020 Oct 22;9(11):3383. doi: 10.3390/jcm9113383 (PMC7690255; doi:10.3390/jcm9113383)
Supplement: Supplementary file 1 [file jcm-09-03383-s001.zip › supplementary materials 3/Table S10.docx]

**Table S10.** Reperfusion therapy impact on MTT ASPECTS versus follow-up ASPECTS

| BV ASPECTS in relation to follow-up ASPECTS | | | | |
| --- | --- | --- | --- | --- |
| Reperfusion | Agreement | | kappa | U-test  p-value |
|  | t = 0 | t = 2 |  |  |
| No reperfusion | 17% | 83% | 0.111 | .005 |
| Thrombectomy | **18%** | **63%** | **0.133** | <.001 |
| Fibrinolysis | 23% | **77%** | **0.360** | .016 |
| Fibrinolysis and thrombectomy | 12% | **72%** | **0.080** | <.001 |
| Thrombectomy without fibrinolysis | **25%** | 54% | **0.196** | .002 |
| Fibrinolysis without thrombectomy | 32% | **82%** | 0.437 | .080* |
| Overall | 22% | **73%** | 0.278 | <.001 |

Best results across tables S9-S12 are bolded

*The only result not exhibiting negative shift
