# Supplementary material for: Real-World Comparison of Human and Software Image Assessment in Acute Ischemic Stroke Patients’ Qualification for Reperfusion Treatment
Source: J Clin Med. 2020 Oct 22;9(11):3383. doi: 10.3390/jcm9113383 (PMC7690255; doi:10.3390/jcm9113383)
Supplement: Supplementary file 1 [file jcm-09-03383-s001.zip › supplementary materials 3/Table S11.docx]

**Table S11.** Reperfusion therapy impact on TMAX ASPECTS versus follow-up ASPECTS

| TMAX ASPECTS in relation to follow-up ASPECTS | | | | |
| --- | --- | --- | --- | --- |
| Reperfusion | Agreement | | kappa | U-test  p-value |
|  | t = 0 | t = 2 |  |  |
| No reperfusion | 39% | **87%** | **0.609** | .356* |
| Thrombectomy | 10% | 55% | 0.083 | < .001 |
| Fibrinolysis | 21% | 62% | 0.241 | < .001 |
| Fibrinolysis and thrombectomy | 12% | 64% | 0.066 | .001 |
| Thrombectomy without fibrinolysis | 8% | 46% | 0.047 | .001 |
| Fibrinolysis without thrombectomy | 29% | 61% | 0.308 | .015 |
| Overall | 22% | 64% | 0.249 | < .001 |

Best results across tables S9-S12 are bolded

*The only result not exhibiting negative shift
