# Supplementary material for: Real-World Comparison of Human and Software Image Assessment in Acute Ischemic Stroke Patients’ Qualification for Reperfusion Treatment
Source: J Clin Med. 2020 Oct 22;9(11):3383. doi: 10.3390/jcm9113383 (PMC7690255; doi:10.3390/jcm9113383)
Supplement: Supplementary file 1 [file jcm-09-03383-s001.zip › supplementary materials 3/Table S12.docx]

**Table S12.** Reperfusion therapy impact on MTT ASPECTS versus follow-up ASPECTS

| MTT ASPECTS in relation to follow-up ASPECTS | | | | |
| --- | --- | --- | --- | --- |
| Reperfusion | Agreement | | kappa | U-test  p-value |
|  | t = 0 | t = 2 |  |  |
| No reperfusion | 17% | 65% | 0.28 | 1* |
| Thrombectomy | 8% | 41% | 0.07 | < .001 |
| Fibrinolysis | 23% | 57% | 0.241 | < .001 |
| Fibrinolysis and thrombectomy | 8% | 32% | 0.066 | .001 |
| Thrombectomy without fibrinolysis | 8% | 50% | 0.074 | < .001 |
| Fibrinolysis without thrombectomy | 36% | 79% | 0.429 | .010 |
| Overall | 18% | 57% | 0.198 | < .001 |

*The only result not exhibiting negative shift
