# Supplementary material for: Real-World Comparison of Human and Software Image Assessment in Acute Ischemic Stroke Patients’ Qualification for Reperfusion Treatment
Source: J Clin Med. 2020 Oct 22;9(11):3383. doi: 10.3390/jcm9113383 (PMC7690255; doi:10.3390/jcm9113383)
Supplement: Supplementary file 1 [file jcm-09-03383-s001.zip › supplementary materials 3/Table S2.docx]

**Table S2.** Reperfusion therapy impact on automatic versus follow-up ASPECTS

| RAPID ASPECTS assessment | | | | |
| --- | --- | --- | --- | --- |
| Reperfusion | Agreement | | kappa | U-test  p-value |
|  | t = 0 | t = 2 |  |  |
| No reperfusion | 30 | 83 | 0.33 | .015 |
| Thrombectomy | 22 | 65 | 0.097 | < .001 |
| Fibrinolysis | 26 | 69 | 0.293 | .088 |
| Fibrinolysis and thrombectomy | 24 | 68 | –0.045 | .132 |
| Thrombectomy without fibrinolysis | 21 | 63 | 0.149 | .002 |
| Fibrinolysis without thrombectomy | 29 | 71 | 0.403 | .397 |
| Overall | 26 | 71 | 0.29 | < .001 |

In all cases of statistically significant differences, a negative shift was detected: follow-up ASPECTS scores were lower than baseline.
