# Supplementary material for: Real-World Comparison of Human and Software Image Assessment in Acute Ischemic Stroke Patients’ Qualification for Reperfusion Treatment
Source: J Clin Med. 2020 Oct 22;9(11):3383. doi: 10.3390/jcm9113383 (PMC7690255; doi:10.3390/jcm9113383)
Supplement: Supplementary file 1 [file jcm-09-03383-s001.zip › supplementary materials 3/Table S4.docx]

**Table S4.** Correlation between ASPECTS and time from symptom onset to CT

| Feature | Correlation coefficient | p-value |
| --- | --- | --- |
| Manual baseline | –0.256 | .01 |
| Automatic baseline | –0.158 | .117 |
| Follow-up | –0.179 | .07 |
| Difference between follow-up and manual baseline | 0.037 | .714 |
| Difference between follow-up and automatic baseline | –0.099 | .323 |
