# Supplementary material for: Real-World Comparison of Human and Software Image Assessment in Acute Ischemic Stroke Patients’ Qualification for Reperfusion Treatment
Source: J Clin Med. 2020 Oct 22;9(11):3383. doi: 10.3390/jcm9113383 (PMC7690255; doi:10.3390/jcm9113383)
Supplement: Supplementary file 1 [file jcm-09-03383-s001.zip › supplementary materials 3/Table S5.docx]

**Table S5.** Agreement of global RAPID ASPECTS scores relative to 6 for raters with percentages of agreement equal or less than 75%

| Rater number | Years of experience | Number of patients | Percentage of agreement | kappa | p-value |
| --- | --- | --- | --- | --- | --- |
| 5 | 28 | 7 | 71% | 0.417 | NA |
| 8 | 8 | 11 | 63.6% | 0.251 | .025 |
| 9 | 23 | 12 | 66.7% | 0.273 | NA |
| 10 | 10 | 16 | 75% | 0.429 | NA |
| 11 | 11 | 18 | 67% | 0.169 | .212 |
