# Supplementary material for: Real-World Comparison of Human and Software Image Assessment in Acute Ischemic Stroke Patients’ Qualification for Reperfusion Treatment
Source: J Clin Med. 2020 Oct 22;9(11):3383. doi: 10.3390/jcm9113383 (PMC7690255; doi:10.3390/jcm9113383)
Supplement: Supplementary file 1 [file jcm-09-03383-s001.zip › supplementary materials 3/Table S6.docx]

**Table S6.** Agreement and correlation between perfusion volumes acquired automatically and manually

| Pair of maps | ICC | | Correlation | |
| --- | --- | --- | --- | --- |
|  | Coefficient | p-value | Coefficient | p-value |
| RAPID CBF – manual CBV | 0.17 (–0.07–0.04) | .102 | **0.387** | **< .001** |
| RAPID TMAX – manual MTT | 0.054 (–0.051–0.187) | .199 | **0.292** | **.004** |
| RAPID TMAX – manual TMAX | 0.11 (–0.072–0.325) | .17 | **0.407** | **< .001** |
| Manual MTT – manual TMAX | **0.903** | **< .001** | **0.941** | **< .001** |

Bold numbers denote statistically significant p-values
