# Supplementary material for: Real-World Comparison of Human and Software Image Assessment in Acute Ischemic Stroke Patients’ Qualification for Reperfusion Treatment
Source: J Clin Med. 2020 Oct 22;9(11):3383. doi: 10.3390/jcm9113383 (PMC7690255; doi:10.3390/jcm9113383)
Supplement: Supplementary file 1 [file jcm-09-03383-s001.zip › supplementary materials 3/Table S7.docx]

**Table S7.** Qualification results comparison chart

| Reperfusion qualification method | Number qualified | DEFUSE 3 | | DAWN | |
| --- | --- | --- | --- | --- | --- |
|  |  | % | kappa | % | kappa |
| DEFUSE 3 | 73 | 100 | **1** | 70 | **0.426** |
| DAWN | 45 | 70 | **0.426** | 100 | **1** |
| Local protocol | 58 | 63 | **0.201** | 55 | 0.114 |
| DEFUSE 3 criteria with manual core and penumbra detection | 30 | 53 | **0.206** | 61 | **0.187** |
| DAWN criteria with manual core detection | 7 | 34 | 0.054 | 60 | **0.125** |

Bold numbers denote statistically significant p-values

Among volumetric parameters, only manually assessed MTT and TMAX presented normal distribution, whereas measurements of the remaining parameters revealed a strong tendency towards lower values.
