# Supplementary material for: Real-World Comparison of Human and Software Image Assessment in Acute Ischemic Stroke Patients’ Qualification for Reperfusion Treatment
Source: J Clin Med. 2020 Oct 22;9(11):3383. doi: 10.3390/jcm9113383 (PMC7690255; doi:10.3390/jcm9113383)
Supplement: Supplementary file 1 [file jcm-09-03383-s001.zip › supplementary materials 3/Table S8.docx]

**Table S8.** Reperfusion therapy impact on CTP concordance with follow-up

| Spearman correlation coefficient rho of measured volume and follow-up ASPECTS | | | | | |
| --- | --- | --- | --- | --- | --- |
| Reperfusion | RAPID CBF | RAPID TMAX | Manual TMAX | Manual MTT | Manual CBV |
| No reperfusion | **–0.862** | **–0.701** | –0.233 | –0.108 | **–0.573** |
| Thrombectomy | **–0.357** | –0.260 | –0.058 | –0.113 | –0.016 |
| Fibrinolysis | **–0.624** | **–0.401** | –0.048 | 0.019 | –0.120 |
| Fibrinolysis and thrombectomy | –0.353 | –0.076 | 0.259 | 0.078 | 0.013 |
| Thrombectomy without fibrinolysis | **–0.461** | **–0.433** | –0.365 | –0.316 | –0.037 |
| Fibrinolysis without thrombectomy | **–0.757** | **–0.566** | –0.052 | 0.019 | –0.272 |
| Overall | **–0.686** | **–0.486** | –0.114 | –0.060 | **–0.236** |

Bold numbers indicate p-values below .05.
