# Supplementary material for: Real-World Comparison of Human and Software Image Assessment in Acute Ischemic Stroke Patients’ Qualification for Reperfusion Treatment
Source: J Clin Med. 2020 Oct 22;9(11):3383. doi: 10.3390/jcm9113383 (PMC7690255; doi:10.3390/jcm9113383)
Supplement: Supplementary file 1 [file jcm-09-03383-s001.zip › supplementary materials 3/Table S9.docx]

**Table S9.** Reperfusion therapy impact on CBF ASPECTS versus follow-up ASPECTS

| CBF ASPECTS in relation to follow-up ASPECTS | | | | |
| --- | --- | --- | --- | --- |
| Reperfusion | Agreement | | kappa | U-test  p-value |
|  | t = 0 | t = 2 |  |  |
| No reperfusion | **44%** | 78% | 0.486 | .944* |
| Thrombectomy | 16% | **63%** | 0.105 | < .001 |
| Fibrinolysis | **30%** | 76% | 0.309 | .002 |
| Fibrinolysis and thrombectomy | **16%** | 68% | 0.064 | .019 |
| Thrombectomy without fibrinolysis | 17% | **58%** | 0.093 | .002 |
| Fibrinolysis without thrombectomy | **43%** | **82%** | **0.459** | .086 |
| Overall | **30%** | 72% | **0.321** | < .001 |

Best results across tables S9-S12 are bolded

*The only result not exhibiting negative shift
