# Supplementary figures and images for: Real-World Comparison of Human and Software Image Assessment in Acute Ischemic Stroke Patients’ Qualification for Reperfusion Treatment
Source: J Clin Med. 2020 Oct 22;9(11):3383. doi: 10.3390/jcm9113383 (PMC7690255; doi:10.3390/jcm9113383)

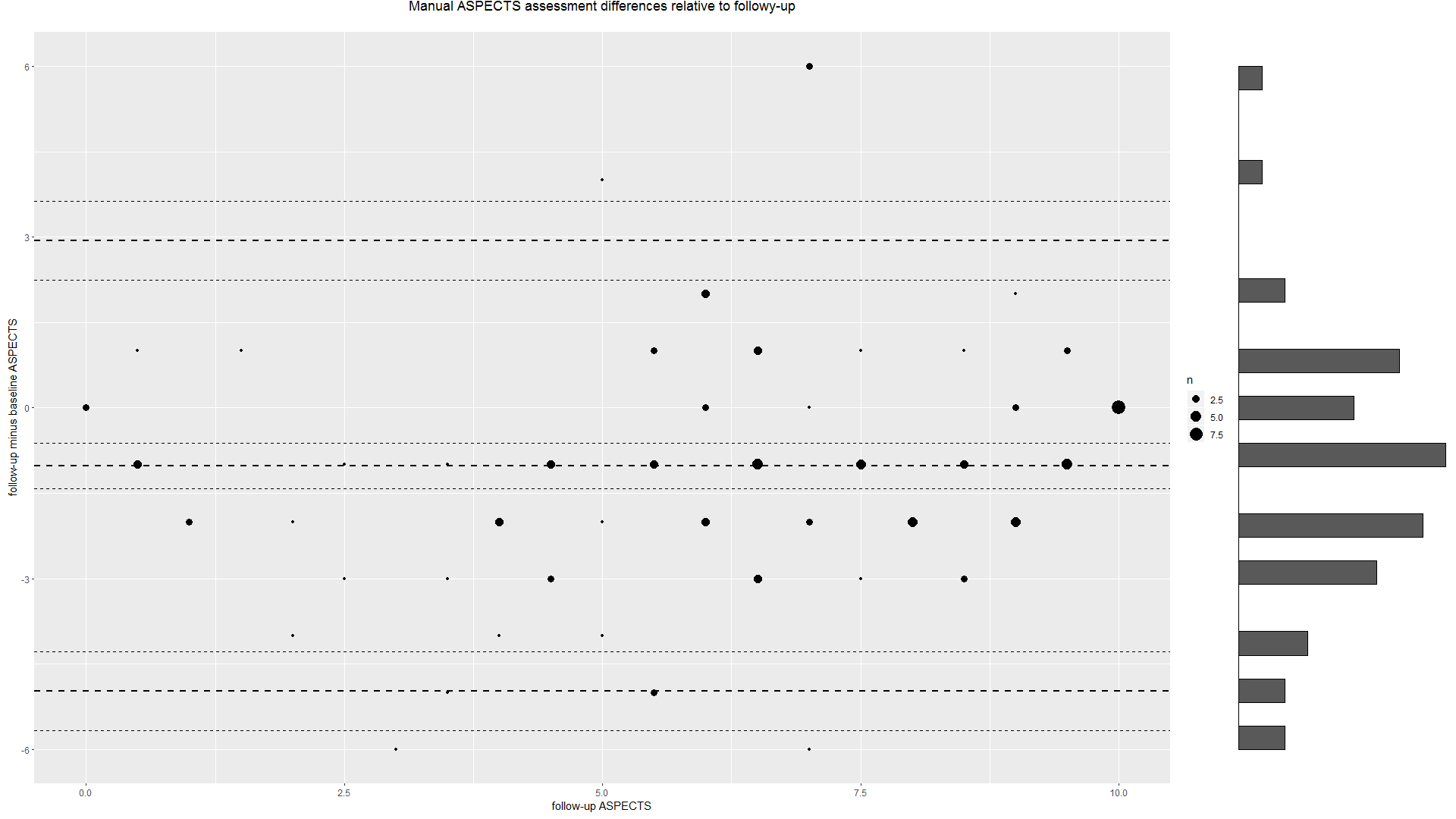

Supplement: Supplementary file 1 [file jcm-09-03383-s001.zip › supplementary materials 3/Figure S1.png]

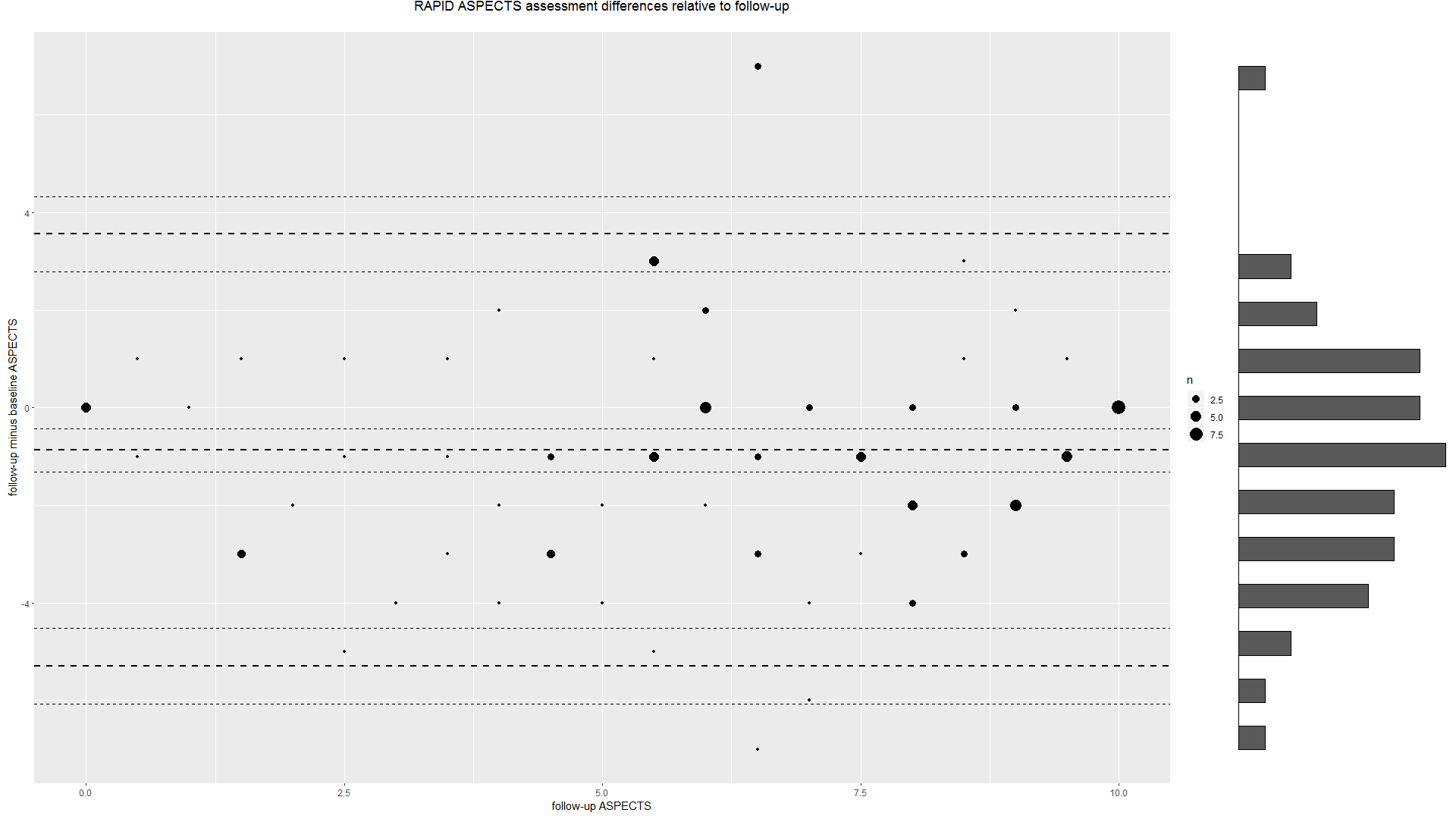

Supplement: Supplementary file 1 [file jcm-09-03383-s001.zip › supplementary materials 3/Figure S2.png]

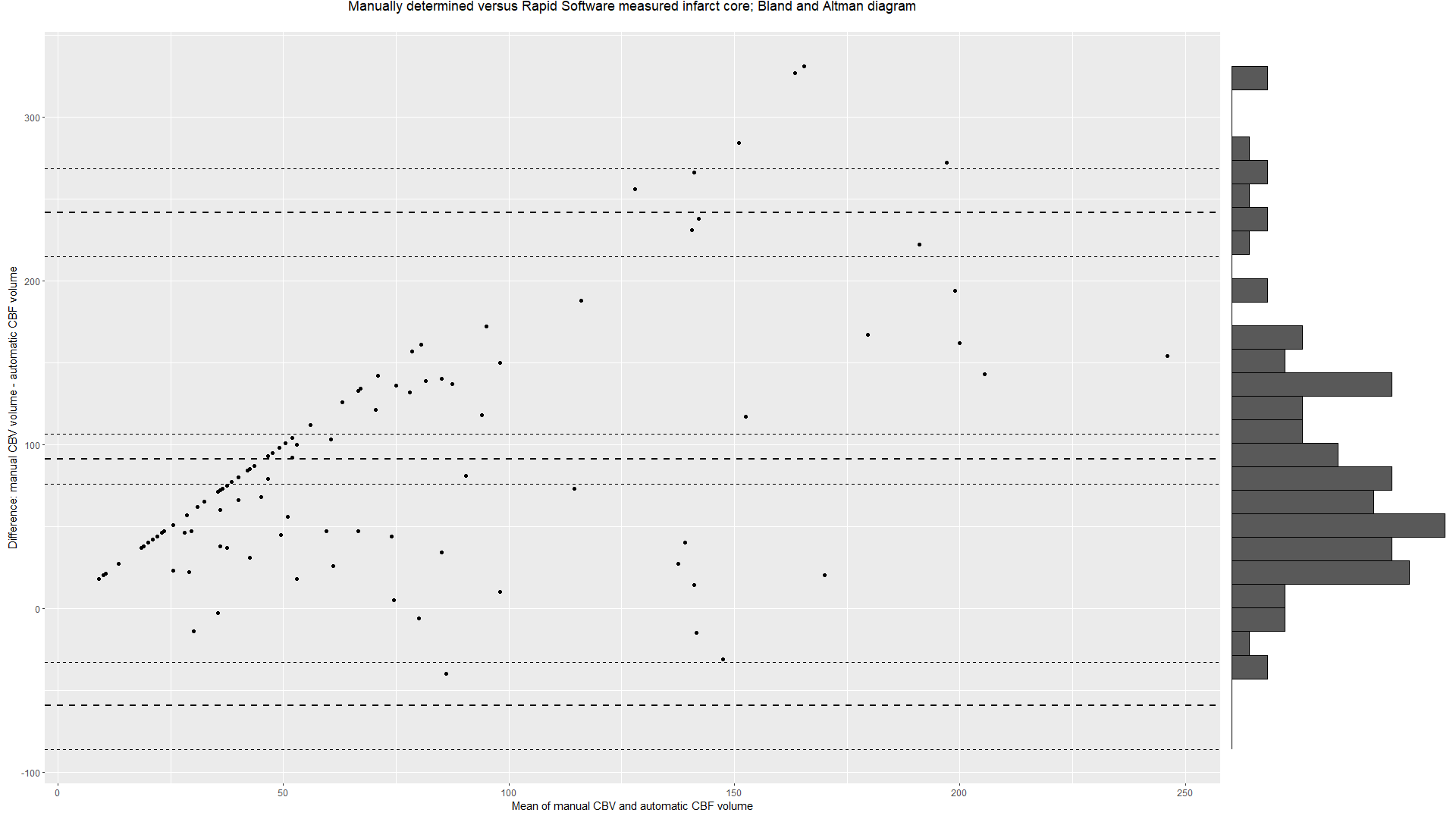

Supplement: Supplementary file 1 [file jcm-09-03383-s001.zip › supplementary materials 3/Figure S3.png]

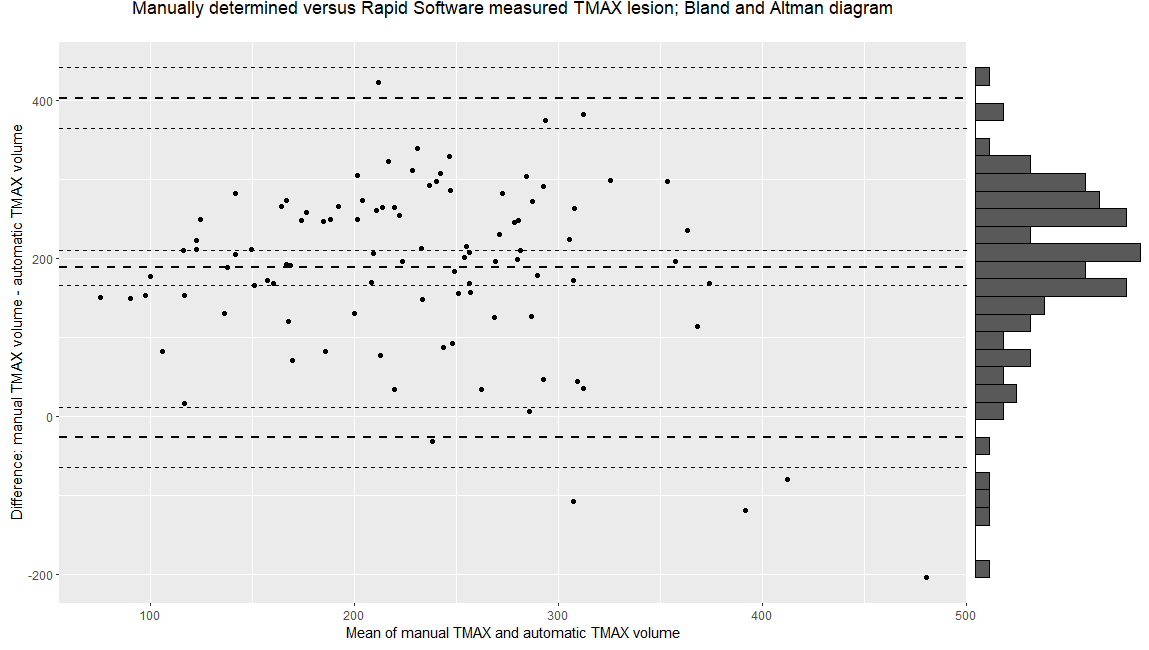

Supplement: Supplementary file 1 [file jcm-09-03383-s001.zip › supplementary materials 3/Figure S4.png]

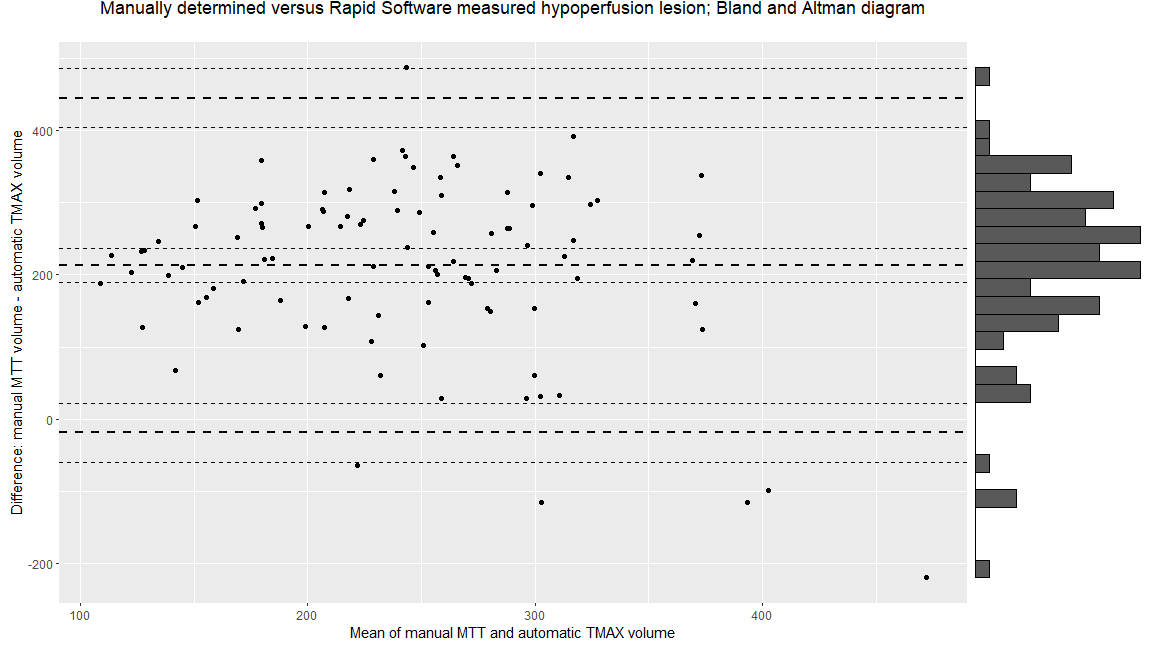

Supplement: Supplementary file 1 [file jcm-09-03383-s001.zip › supplementary materials 3/Figure S5.png]

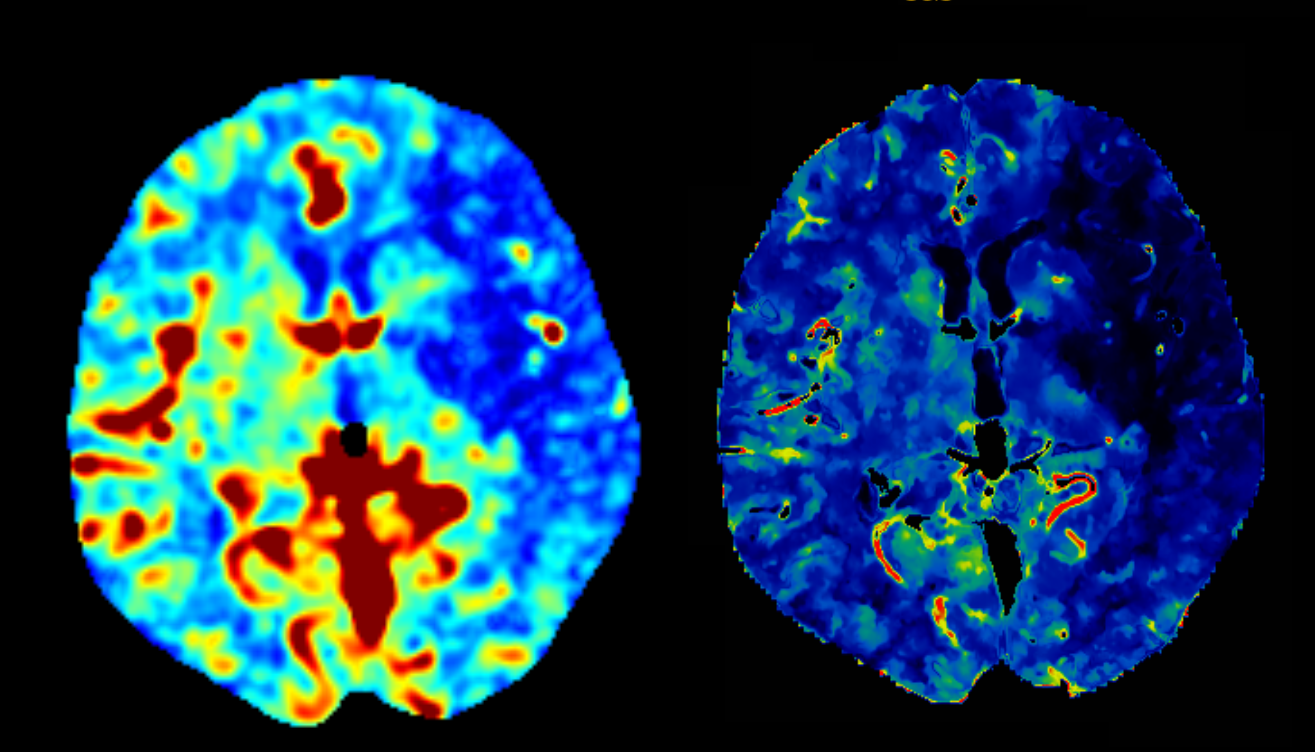

Supplement: Supplementary file 1 [file jcm-09-03383-s001.zip › supplementary materials 3/Figure S6.png]
